# We Still Have to Fear Malaria: A Case Report of Severe Malaria With Almost all the Listed WHO Complications in a Patient Living in a Sub‐Saharan Endemic Area

**DOI:** 10.1155/crdi/7246677

**Published:** 2025-12-17

**Authors:** Sylvain Raoul Simeni Njonnou, Martial Tsiazok Dongmo, Loic Oleg Djilo Kouonang, Borice Tsafack Tapondjou, Erwan Ndembe Djeni, Siméon Pierre Choukem

**Affiliations:** ^1^ Department of Internal Medicine and Specialities, Faculty of Medicine and Pharmaceutical Sciences, University of Dschang, Dschang, Cameroon, univ-dschang.org; ^2^ Department of Internal Medicine, Dschang Regional Annex Hospital, Dschang, Cameroon; ^3^ Health and Human Development (2HD) Research Network, Douala, Cameroon; ^4^ Department of Nephrology, Bafoussam Regional Hospital, Bafoussam, Cameroon; ^5^ Department of Chemistry, Faculty of Science, University of Dschang, Dschang, Cameroon, univ-dschang.org; ^6^ Higher Institute of Health Sciences, Université des Montagnes, Bangangté, Cameroon, udesmontagnes.org; ^7^ Department of Internal Medicine, Douala General Hospital, Douala, Cameroon

**Keywords:** acute kidney injury, hypoglycemia, liver failure, respiratory failure, severe malaria, status epilepticus

## Abstract

**Background:**

Malaria is a common and potentially deadly infection in Sub‐Saharan Africa, causing nearly 600,000 deaths. Despite improvements in treatment and prevention, it continues to wreak havoc, particularly in this region, which accounts for more than 95% of cases. Individuals living in malaria‐endemic areas traditionally have a lower risk of developing severe malaria.

**Case Presentation:**

We report a case of a 67‐year‐old woman with hypertension and bilateral knee osteoarthritis. She was referred from a health center due to confusion and abnormal movements in a febrile context. Findings upon admission revealed a patient with a poor general state, an inflammatory syndrome, a confusional syndrome, a cortical irritation syndrome, a hemolytic anemia, and a severe hypoglycemia. Both the rapid diagnostic test and the thick blood smear for malaria were positive. The clinical course was marked by persistent signs of hemolysis and hypoglycemia, status epilepticus, deep coma, the development of diffuse ecchymoses, digital ischemia and Stage 2 pressure ulcers, worsening of respiratory failure, hepatocellular failure, acute kidney injury, and hyperkalemia reaching 6.24 mmol/L. Therapeutic interventions led to significant improvements in the patient’s level of consciousness, resolution of status epilepticus, correction of hypoglycemia, and attenuation of hemolysis, although acute kidney injury persisted, requiring extrarenal epuration. Despite improvements in consciousness and correction of respiratory, liver, and kidney function, the patient ultimately succumbed to sepsis before digital amputation could be performed.

**Conclusion:**

This case serves as a reminder of the severe complications and potential fatality associated with malaria. Emphasis must be placed on prevention.

## 1. Introduction

Malaria is a parasitic infection caused by *Plasmodium* spp and is responsible for morbidity and mortality, particularly in areas where it is endemic [1, 2]. It is transmitted by mosquitoes of the genus *Anopheles*, particularly the female, during her meal. The *Plasmodium* parasite has a multistage lifecycle, which leads to characteristic cyclical fevers [3]. Despite significant advances in diagnosis (e.g., PCR) and treatment (e.g., new antimalarial drugs and antimalarial vaccines), this disease remains a major public health problem in Sub‐Saharan Africa [2, 4]. It is responsible for 263 million cases and almost 600,000 deaths in 2023, most of them (over 95%) in Sub‐Saharan Africa [5]. Malarial complications include different types of manifestations (clinical and biological), ranging from metabolic to clinical or biological. Several systems may be involved: hematologic (anemia and jaundice, bleeding), cardiovascular (shock), respiratory (respiratory distress), urogenital (hemoglobinuria, acute kidney injury), digestive (vomiting), and neurological (convulsion, coma/alteration of consciousness) [6, 7].

Antimalarial immunity refers to the body’s ability to resist or overcome a malaria infection. It is a complex and evolving process that involves both humoral and cellular immune responses. It could be influenced by several parameters depending on the host (age and comorbidities) and the parasite (exposure and type) [8]. Individuals living in a malaria‐endemic region may develop a form of immunity through repeated exposure, which is principally mediated by antibodies [9]. However, this immunity is not always protective against severe malaria, particularly in children or adults with recent exposure or immunity‐reducing disease [10]. It is nevertheless surprising to find an individual living in an endemic zone with no known reducing‐immunity disease, who develops several serious complications of malaria. We present the case of a 67‐year‐old African lady who has always lived in this area and does not have any immunocompromised disease, who has been admitted for severe malaria. She presented almost all the severity criteria of malaria.

## 2. Case Presentation

A 67‐year‐old black woman, living with hypertension (treated with nifedipine) and knee osteoarthritis, was referred from a health center for confusion and abnormal movements in a febrile context. She had been experiencing, for 2 days, progressive asthenia, headaches, fever, and two episodes of vomiting. This prompted her to take an undocumented antimalarial drug. Given the persistence of symptoms, she consulted in a nearby health facility where a rapid malaria test was performed (positive), and she was treated with IV artesunate 2.6 mg/kg. Evolution was marked by the occurrence of a progressive confusional state and seizures of the generalized tonic–clonic type (2 episodes) that prompted the reference to our health facility (a second‐line health facility of a semiurban area).

The clinical exam at the entrance revealed the following: an asthenic patient with sclerotic and cutaneous jaundice and normal blood pressure (BP) (125/77 mmHg); systemic inflammatory response syndrome (T° 38.9°C, *π* = 113 bpm); confusional syndrome (GCS: 13/15), and cortical irritation syndrome (the patient presented a two‐minute tonic–clonic seizure without sphincter relaxation or tongue bite), without meningeal or focal signs, clinical anemia, and hypoglycemia (0.6 g/L). The rapid test for malaria was positive, same as the thick smear (with a parasitemia of 67 trophozoites/mm^3^). Blood cultures were performed and were negative. The treatment with artesunate was therefore continued with the adjunction of glucose 10% drips.

Patient presented 2 hours later with a status epilepticus associated with deep alteration of consciousness (GCS: 3/15) without meningeal or focal signs in the setting of persistent hypoglycemia and respiratory failure (RR: 36 cpm, SPO_2_: 65% on ambient air, blood gases not performed). She was admitted to the intensive care unit, where respiratory support, correction of hypoglycemia, and treatment of status epilepticus (with clonazepam and phenobarbital) were performed.

Biological workup at entrance revealed the following: severe thrombopenia (PLT: 24 G/L, normal range: 150–350 G/L), hemolytic anemia (Hb: 7.6 g/dL [normal range: 12–16 g/dL], normocytic normochromic with total bilirubin 3.1 mg/dL [normal range: < 1.0 mg/dL], and conjugated bilirubin 0.3 mg/dL [normal range: < 0.2 mg/dL]), C‐reactive protein (CRP) (96 mg/L, normal range: ≤ 6 mg/L), cytolysis (GOT/GPT: 56/67 UI/L, normal range: 36/40 UI/L), acute kidney injury (blood urea/serum creatinine: 128.8 mg/dL/1.78 mg/dL, normal range: 10–40 mg/dL/0.5–1.10 mg/dL), and moderate hyponatremia (Na^+^: 127.6, normal range: 135–155 mmol/L) with normal kalemia (K+: 5.44, normal range: 3.5–5.5 mmol/L), normal prothrombin time (PT) (76%, normal range: > 70%), and albumin (38 g/L, normal range: 36–40 g/L) (Table 1).

**Table 1:** Biological characteristics of the reported case and evolution.

Biological parameters	At entrance	48 h later	At the referral time	Before death	Normal range
Malaria rapid diagnosis test	Positive				Negative
Thick smear	67 Trophozoites/mm^3^				Negative
Hemoglobin (Hb)	7.6	10.1	8.8	9.1	12–16 g/dL
Platelet (PLT)	20	66	114	132	150–350 G/L
Total bilirubin	3.1		1.57		< 1.0 mg/dL
Conjugated bilirubin	0.3		0.64		< 0.2 mg/dL
CRP	96	6	24	355	≤ 6 mg/L
GOT	56	405	139	46	36 UI/L
GPT	67	221	216	32	40 UI/L
Blood urea	128.8	197	337	143	10–40 mg/dL
Serum creatinine	1.78	3.46	8.9	2.4	0.5–1.10 mg/dL
Na+	127.6	138.1	139.9	140	135–155 mmol/L
K+	5.44	6.24	7.09	4.6	3.5–5.5 mmol/L
Prothrombin time (PT)	76%	40%	90%	90%	> 70%
Albumin	38				36–40 g/L
APTT		48 s			20.6–28.6 s
Blood culture	Sterile			Negative	Negative
Urine culture	Sterile			Negative	Negative
Creatinine phosphokinase (CPK)			3031	1034	30–150 UI/L

**Figure 1:**
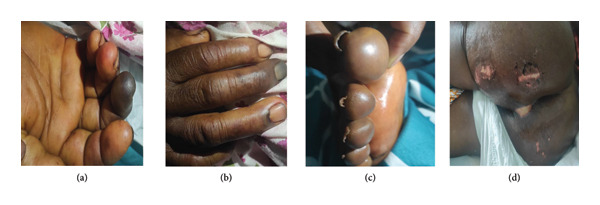
Digital ischemia on the fingers and toes (a, b, c) and Stage 2 pressure ulcers (d).

Evolution was marked by persistent signs of hemolysis (requiring blood transfusion) and hypoglycemia, status epilepticus, coma (GCS: 3/15), the appearance of diffuse ecchymotic lesions and digital ischemia (Figure 1) with prolonged activated partial thromboplastin time (APTT) (48 s, normal range: 23–38 s), the appearance of Stage 2 pressure ulcers, increased respiratory failure, hepatocellular insufficiency (PT: 40%, GOT/GPT: 405/221 UI/L) and increased AKI (urea: 197 mg/dL, serum creatinine: 3.46 mg/dL, and oliguria: 450 mL/24 h), and the occurrence of hyperkalemia at 6.24 mmol/L. This prompted us to add intravenous (IV) vitamin K 10 mg at once, IV calcium gluconate, and corticosteroids (methylprednisolone 1 mg/kg/day for 3 days) to her management.

**Figure 2:**
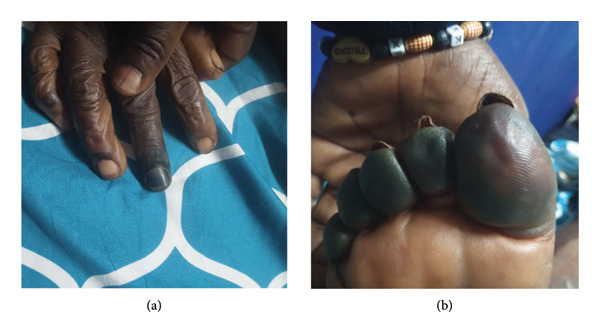
Worsening of digital ischemia on the fingers and toes (a and b).

Finally, the patient presented a clear improvement in the state of consciousness (GCS 11/15 M5V2Y4); fever disappearance (T° 36.7°C); a reduction in oxygen requirements; correction of the hypoglycemia; reduction in cytolysis (GOT/GPT: 219.4/119.6 UI/L) and thrombopenia (PLT 80 G/L), but worsening renal function with major hyperkalemia (urea 337 mg/dL, serum creatinine 8.9 mg/dL; K+ 7.07 mmol/L) and severe rhabdomyolysis (CPK: 3031 UI/L, normal range: 30–150 UI/L); and aggravation of digital ischemia motivating referral to the Bafoussam Regional Hospital. There, she underwent three sessions of extrarenal purification by hemodialysis with resumption of diuresis, reduction of rhabdomyolysis (1304 UI/L), and normalization of consciousness (GCS 15/15) but worsening of digital ischemia and resurgence of fever (in the setting of high inflammatory syndrome: CRP 355 mg/L) (Figure 2). This was suggestive of an infectious process requiring a bacteriological workup (negative hemoculture and urine culture), and antibiotherapy with ceftriaxone and levofloxacin was started. The Doppler ultrasound of the lower and upper limbs showed complete thrombosis of the medial and lateral digital arteries of the left hand and right foot, indicating amputation of certain fingers and toes. The patient presented a hemodynamic imbalance during the 6^th^ hemodialysis session before amputation, leading to dialysis cessation, and she had to be resuscitated. However, she died within a few hours.

## 3. Discussion

We describe the symptomatology of a 67‐year‐old patient from Sub‐Saharan Africa living in an endemic area for malaria who had been experiencing asthenia, headaches, vomiting, and progressive altered level of consciousness in a febrile context. Upon admission, she displayed neurological symptoms (in addition to confusion and multiple seizures without meningeal irritation) and signs consistent with hemolytic anemia. Based on all these clinical and workup findings, a diagnosis of severe malaria was made. It is challenging to rule out the possibility of concurrent coinfections by other bacteria or viruses (such as dengue or CMV, or chikungunya) due to this wide range of clinical manifestations [11]. However, it is important to remember that Sub‐Saharan African countries (especially in rural areas) have limited access to investigations, particularly in terms of bacteriology and specific imaging such as CT‐scan or MRI [12]. This situation was similar during the COVID‐19 pandemic, also accompanied by the lack of insurance, leading people to pay for themselves [13].

Dengue fever and chikungunya may be listed as malaria‐associated infections in this patient because they circulate in the same geographical area and have the same vectors [14]. However, malaria alone, could, possibly be responsible for all these symptoms. A rare malaria complication could possibly lead to this dramatic status: hemophagocytosis. The presence of APTT prolongation, as well as cytopenias and cytolysis, would suggest this pathology [15, 16]. Unfortunately, we did not perform the dosage of triglycerides, and the technical facilities of the health facilities did not allow us to perform a myelogram, as well as ferritin and fibrinogen. Another argument in favor of this diagnosis was the patient’s improvement after adding corticosteroids.

In the end, we were extremely surprised by the severity of the clinical picture (which we attributed to malaria) in this patient. Apart from the acidosis (which, however, should have been present because of the uremia and possibly the respiratory failure), the hyperparasitemia, and the shock, this patient presented with all the complications of malaria listed by the WHO [5]. Moreover, hyperparasitemia is frequently absent in the neurological manifestations of malaria because of the encephalitic sequestration of the parasite [17]. Although the prevalence of malaria is higher in rural than in urban areas in Cameroon, we did not expect such a large number of severe complications [18]. This patient’s antimalarial immunity should have enabled her to have either a mild or less severe infection. In any case, the intensity of the infestation and the state of her immune defenses predispose her to malaria complications [8].

The patient’s death was probably due to septic shock. Bacteriological cultures were negative, but there was a clinical and biological inflammatory syndrome. One of the most likely sources of infection in this patient was a dialysis catheter infection, but there was also bacterial endocarditis (despite the absence of a murmur).

The main limitation in the presentation of this case report is the lack of certain tests that would have enabled us to rule out other viral coinfections (dengue, chikungunya, and other arboviruses) and the possible complication of hemophagocytosis.

## 4. Conclusion

Malaria, even though frequently diagnosed, still presents with diverse clinical manifestations, especially in severe cases. This diversity of signs, most of the time, equally presents great issues of differential and coinfection diagnosis, especially in Sub‐Saharan Africa, where financial means for investigations are not always available for the majority. However, rapid diagnosis and management, as well as preventive measures, are the key to improving patient outcomes.

**Nomenclature**
CRP: C‐Reactive protein
CPK: Creatinine phosphokinase
GCS: Glasgow Coma Scale
GOT: Glutamate oxaloacetic transaminase
GPT: Glutamate pyruvate transaminase
Hb: Hemoglobin
IV: Intravenous
PCR: Polymerase chain reaction
PLT: Platelet
RR: Respiratory rate
SPO_2_
: Saturation pulsée en oxygène (pulsed oxygen saturation)

## Ethics Statement

Ethical approval was not required for a single‐patient case report.

## Consent

The family members approved the case report and image publication.

## Disclosure

This case was presented as a poster at the 1^st^ scientific congress of the Cameroonian Society of Internal Medicine from the 2^nd^ to 4^th^ October 2025 in Yaoundé. All authors read and approved the final version for publication.

## Conflicts of Interest

The authors declare no conflicts of interest.

## Author Contributions

Design and design: Sylvain Raoul Simeni Njonnou, Martial Tsiazok Dongmo, and Siméon Pierre Choukem.

Data collection: Sylvain Raoul Simeni Njonnou, Martial Tsiazok Dongmo, Loic Oleg Djilo Kouonang, and Borice Tsafack Tapondjou.

Patient management: Sylvain Raoul Simeni Njonnou, Loic Oleg Djilo Kouonang, and Martial Tsiazok Dongmo.

Manuscript writing: Erwan Ndembe Djeni.

Manuscript revision: Sylvain Raoul Simeni Njonnou, Martial Tsiazok Dongmo, and Siméon Pierre Choukem.

## Funding

No funding was received for this study.

## Data Availability

The data that support the findings of this study are available on request from the corresponding author. The data are not publicly available due to privacy or ethical restrictions.

## References

[1] Daily J. P. , Minuti A. , and Khan N. , Diagnosis, Treatment, and Prevention of Malaria in the US: A Review, JAMA. (2022) 328, no. 5, 10.1001/jama.2022.12366. 35916842

[2] Varo R. , Chaccour C. , and Bassat Q. , Update on Malaria, Medical Clinics of North America. (2020) 155, no. 9, 395–402, 10.1016/j.medcle.2020.05.024. 32620355

[3] Moxon C. A. , Gibbins M. P. , McGuinness D. , Milner D. A. , and Marti M. , New Insights Into Malaria Pathogenesis, Annual Review of Pathology: Mechanisms of Disease. (2020) 15, no. 1, 315–343, 10.1146/annurev-pathmechdis-012419-032640. 31648610

[4] Shahbodaghi S. D. and Rathjen N. A. , Malaria: Prevention, Diagnosis, and Treatment, American Family Physician. (2022) 106, no. 3, 270–278. 36126008

[5] Venkatesan P. , WHO World Malaria Report 2024, The Lancet Microbe. (2025) 6, no. 4, https://www.thelancet.com/journals/lanmic/article/PIIS2666-5247(25)00001-1/fulltext, 10.1016/j.lanmic.2025.101073. 39923782

[6] Balaji S. , Deshmukh R. , and Trivedi V. , Severe Malaria: Biology, Clinical Manifestation, Pathogenesis and Consequences, Journal of Vector Borne Diseases. (2020) 57, no. 1, 10.4103/0972-9062.308793. 33818449

[7] White N. J. , Severe Malaria, Malaria Journal. (2022) 21, no. 1, 10.1186/s12936-022-04301-8. PMC9536054 36203155

[8] Artavanis-Tsakonas K. , Tongren J. E. , and Riley E. M. , The War Between the Malaria Parasite and the Immune System: Immunity, Immunoregulation and Immunopathology, Clinical and Experimental Immunology. (2003) 133, no. 2, 145–152, 10.1046/j.1365-2249.2003.02174.x, 2-s2.0-0042667124. 12869017 PMC1808775

[9] Kurup S. P. and Harty J. T. , γδ T Cells and Immunity to Human Malaria in Endemic Regions, Annals of Translational Medicine. (2015) 3, no. Suppl 1, 10.3978/j.issn.2305-5839.2015.02.22, 2-s2.0-85015482525. PMC4437949 26046068

[10] Kwenti T. E. , Moye A. L. , Wiylanyuy A. B. , Njunda L. A. , and Nkuo-Akenji T. , Variation in the Immune Responses Against Plasmodium falciparum Merozoite Surface Protein-1 and Apical Membrane Antigen-1 in Children Residing in the Different Epidemiological Strata of Malaria in Cameroon, Malaria Journal. (2017) 16, no. 1, 10.1186/s12936-017-2105-4, 2-s2.0-85033565243. PMC5679504 29121929

[11] Buck E. and Finnigan N. A. , Malaria. StatPearls [Internet], 2025, StatPearls Publishing, http://www.ncbi.nlm.nih.gov/books/NBK551711/. 31869175

[12] Ge Y. , Liang D. , Cao J. , Gosling R. , Mushi V. , and Huang J. , How Socioeconomic Status Affected the Access to Health Facilities and Malaria Diagnosis in Children Under Five Years: Findings From 19 Sub-Saharan African Countries, Infectious Diseases of Poverty. (2023) 12, no. 1, 10.1186/s40249-023-01075-2. PMC10077698 37024969

[13] Simeni Njonnou S. R. , Kemta Lekpa F. , Ngongang Ouankou C. , Vounsia Balti E. , and Choukem S. P. , The Challenge of COVID-19 Case Identification and Ascertainment in Sub-Saharan Africa: The Case of Cameroon, Pan African Medical Journal. (2020) 35, no. 2, 84–86, 10.11604/pamj.supp.2020.35.24368. PMC7875772 33623608

[14] Djeunang Dongho G. B. , Venturi G. , Fortuna C. et al., Dengue and Chikungunya Virus Circulation in Cameroon and Gabon: Molecular Evidence Among Symptomatic Individuals, Access Microbiology. (2022) 4, no. 4, 10.1099/acmi.0.000340. PMC9260096 35812708

[15] Debaugnies F. , Mahadeb B. , Ferster A. et al., Performances of the H-Score for Diagnosis of Hemophagocytic Lymphohistiocytosis in Adult and Pediatric Patients, American Journal of Clinical Pathology. (2016) 145, no. 6, 862–870, 10.1093/ajcp/aqw076, 2-s2.0-85013689957. 27298397

[16] Simeni Njonnou S. R. , Couturier B. , Gombeir Y. et al., Pituitary Gland and Neurological Involvement in a Case of Hemophagocytic Syndrome Revealing an Intravascular Large B-Cell Lymphoma, Case Reports in Hematology. (2019) 2019, 1–6, 10.1155/2019/9625075. PMC6512020 31183225

[17] Akide N. O. B. , Kilian N. , and Salman M. M. , Cerebral Malaria and Neuronal Implications of Plasmodium falciparum Infection: From Mechanisms to Advanced Models, Advanced Science (Weinheim). (2022) 9, no. 36, 10.1002/advs.202202944. PMC9798991 36300890

[18] Doumbe-Belisse P. , Kopya E. , Ngadjeu C. S. et al., Urban Malaria in Sub-Saharan Africa: Dynamic of the Vectorial System and the Entomological Inoculation Rate, Malaria Journal. (2021) 20, no. 1, 10.1186/s12936-021-03891-z. PMC8424958 34493280

